# Thymoquinone against infectious diseases: Perspectives in recent pandemics and future therapeutics

**DOI:** 10.22038/ijbms.2021.56250.12548

**Published:** 2021-08

**Authors:** Mousumi Tania, Asaduzzaman Asad, Tian Li, Md. Shariful Islam, Shad Bin Islam, Md. Munnaf Hossen, Mizanur Rahman Bhuiyan, Md. Asaduzzaman Khan

**Affiliations:** 1 Research Division of Nature Study Society of Bangladesh, Dhaka, Bangladesh; 2 Division of Molecular Cancer, Red Green Research Center, Dhaka, Bangladesh; 3 Department of Biochemistry and Molecular Biology, Jahangirnagar University, Savar, Dhaka, Bangladesh; 4 The Research Center for Preclinical Medicine, Southwest Medical University, Luzhou, Sichuan, China; 5 Department of Biochemistry and Molecular Biology, Tejgaon College, National University, Dhaka, Bangladesh; 6 Bachelor in Medicine and Surgery Program, Affiliated Hospital of Southwest Medical University, Luzhou, Sichuan, China; 7 Department of Immunology, Health Science Center, Shenzhen, University, Shenzhen, Guangdong, China

**Keywords:** Antimicrobial natural- products, Future therapeutics, Infectious disease, SARS-CoV, Thymoquinone

## Abstract

The recent pandemics caused by coronavirus infections have become major challenges in 21^st^ century human health. Scientists are struggling hard to develop a complete cure for infectious diseases, for example, drugs or vaccines against these deadly infectious diseases. We have searched papers on thymoquinone (TQ) and its effects on different infectious diseases in databases like Pubmed, Web of Science, Scopus, and Google Scholar, and reviewed them in this study. To date research suggests that natural products may become a potential therapeutic option for their prodigious anti-viral or anti-microbial effects on infectious diseases. TQ, a natural phytochemical from black seeds, is known for its health-beneficial activities against several diseases, including infections. It is evident from different *in vitro* and *in vivo* studies that TQ is effective against tuberculosis, influenza, dengue, Ebola, Zika, hepatitis, malaria, HIV, and even recent pandemics caused by severe acute respiratory syndrome of coronaviruses (SARS-CoV and SARS-CoV-2). In these cases, the molecular mechanism of TQ is partly clear but mostly obscure. In this review article, we have discussed the role of TQ against different infectious diseases, including COVID-19, and also critically reviewed the future use of TQ use to fight against infectious diseases.

## Introduction

Infectious diseases are generally caused by different microorganisms such as viruses, bacteria, fungi, or parasites. Some of the infectious diseases can be transmitted from human to human while some others are spread by insects or other animals. Some infectious diseases can be a threat to human lives as those are difficult to treat due to their resistance to commonly used drugs (1, 2). More recently, a deadly strain of the coronavirus has emerged that caused a major outbreak of respiratory disease (pneumonia-like) in China at the end of 2019 (3). This infectious disease has been named coronavirus disease 2019 (COVID-19), and its causative agent has been referred to as severe acute respiratory syndrome coronavirus 2 (SARS-CoV-2) (4). 

Natural products are good sources for drug research to develop novel candidates against infectious diseases. Conventional drugs against infectious diseases could manage the major symptoms of the patients easily, but these drugs may cause some side effects. Developing natural products for infection control gained a lot of interest because of fewer side effects, lower cost, and easy access, as well as public approval and promising outcomes (5, 6). Among various medicinal plants, *Nigella sativa* (black seed or black cumin) displayed strong antimicrobial actions. This medicinal plant has been used for years for the treatment of infectious diseases without reported side effects (7, 8). Both black cumin seeds and oil have pharmacological properties and are popularly known as ‘Prophetic medicine’, as the prophet Muhammed (Sm) has described its curative powers in every illness except death. The Persian philosopher and physician Ibn Sina had mentioned it in “Canon of Medicine”, and suggested it as a treatment for several diseases including infectious diseases caused by different pathogens (9, 10). *N. sativa* contains many bioactive components, such as thymoquinone (TQ), alkaloids (nigellicines and nigelledine), sterols and saponins (alpha-hederin), flavonoids, volatile oils of varying composition, lipid constituents and fatty acids, proteins, and many others that have therapeutic properties (11, 12). Among these, the most important component is TQ (chemically, 2-methyl-5-isopropyl-1,4-benzoquinone), which has been identified as a promising natural compound for the treatment of a wide range of diseases, including chronic non-infectious and infectious disease. TQ showed antimicrobial activity against broad-spectrum microorganisms such as viruses, bacteria, and fungi (7, 10, 13). Studies proposed that TQ might prevent SARS-CoV-2 entry and replication inside the host cell, and can modulate immune responses (14, 15). 

In this review, we have discussed the potential anti-infective effects of TQ with possible mechanisms of action and future usage of TQ in clinical applications for the prevention and treatment of infectious diseases. We have searched papers on TQ and its effects on different infectious diseases in databases like Pubmed, Web of Science, Scopus, and Google Scholar. For historical aspects, we had reviewed papers with no time limit, but in this article, we have mostly discussed recent findings. 

***Infectious disease pandemics in 21*** ^st^ ***century: The likelihood of more dreadful reappearance***

Pandemics have always been considered as some of the deadliest threats to human existence that come with apprehension and decimation. We are merely in the 1^st^ quarter of the 21^st^ century but the human existence is crouching through multiple and unfamiliar viral pandemics since 2003 when severe acute respiratory syndrome (SARS) threatened as nearly a pandemic, the first existential threat of this century (16). Since then, we have experienced influenza (H1N1) epidemic in 2009, another coronaviruse epidemic in the Middle East countries (Middle East respiratory syndrome, MERS) in 2012, a chikungunya epidemic in 2014, Zika epidemic in 2015, a widespread epidemic-like extension of Ebola over five African countries from 2014 to 2015, and have been struggling with a novel coronavirus (SARS-CoV-2) in 2019 again (17,18). Before the 21^st^-century, coronaviruses (CoVs) were thought to appear to great significance in veterinary medicine with a very mild effect on humans. However, identifying different CoVs as the etiological agents behind the nearest three pandemics reminds archaic contiguous foes (19). Table 1 summarizes the recent pandemic diseases.

The first incident of SARS was reported in November 2002 in Foshan, Guangdong Province, China, and in April 2003, WHO announced a SARS epidemic with a new CoV as the etiological agent (20, 21). With 95% symptom development and an incubation period of 2-7 days, SARS-CoV has been reported to cause more than 8000 incidents in 30 countries with more than 900 deaths worldwide, where 83% of cases were identified in China. Patients aged over 60 have a mortality rate of 43%, whereas no child death is reported. Healthcare facilitators who seemed highly susceptible accounted for 21% of the cases which is precisely notable (20-22). It was largely postulated that the horseshoe bat works as the reservoir to SARS-CoV and the pathogen then jumped to humans by intermediate hosts like civets or dromedaries. Fortunately, the absence of new infections led to termination declaration of the SARS pandemic in 2004 (23).

A decade after the first CoVs instigated SARS-CoV pandemic, it appeared again as MERS-CoV in September 2012 in Saudi Arabia with higher genetic variation and greater virulent pathogenicity. MERS-CoV disseminated rapidly in dozens of countries mostly in the Middle East where bat acted as the primary reservoir, and dromedary camel was the intermediate host tentatively (23-25). MERS pandemic is not over yet, and by the end of 2020, a total of 2562 laboratory-conﬁrmed cases of MERS including 881 associated deaths were reported globally with a high case fatality ratio of 34.4% (26). 

Frustratingly, in 7 years of MERS, SARS-CoV-2, which lies in the same genus *Betacoronavirus* as SARS-CoV and MERS-CoV, was identified as the etiological agent behind the new zoonotic disease, COVID-19 (27). COVID-19 is a deadly infectious disease, and the World Health Organization (WHO) had declared it a pandemic due to its rapid spread out worldwide (28). At the end of 2020, the total reported cases of COVID-19 were over 83.5 million with 1.8 million deaths worldwide, where the highest number of cases and deaths were reported in the USA. The SARS-CoV-2 virus is highly contagious and spreads by obscure routes of transmission including saliva or respiratory air droplets and with high sustainability in the open air. It indicates mild and asymptomatic pathogenicity along with the ability to adhere to human cells (29, 30). SARS-CoV and SARS-CoV-2 bind with human angiotensin-converting enzyme 2 (ACE2) mostly found in the alveolar cells and other down respiratory tracts. CoVs down-regulate ACE2 and cause pathogenesis in the lung. On the other hand, MERS-CoV binds with human dipeptidyl peptidase 4 (DPP4 or CD26) receptors, which are usually expressed in the upper respiratory tract and alveoli. Dysregulated DPP4 manipulates T-cell mediated inflammations. Although these CoVs are genetically different from each other, their disease manifestations share some common features. They are all from the same genus, use bats as the common primary reservoir, their transmission is through human-animal contacts, their rapid spread takes place through respiratory droplets, the incubation period ranges from 2 to 14 days, very much resemble or overlap the symptomatic demography, and have higher morbidity in aged persons especially those with chronic diseases (31-33).

The major and catastrophic risk revealed from different studies on CoVs is related to their self-evolution and adaptation aptitude. Due to the absence of herd immunity in most people, RNA viruses like CoVs can be more deleterious. Moreover, the high error-prone replication and pliability of mutation under environmental selective pressure enhances its adaptability (18, 34). Co-occurrence and co-existence of human-animal viruses in one host sometimes produce new variants from the re-assortment process of their genetic materials and inflate pandemics. Mutation in the RNA of CoVs can be accumulated, merged with other nucleic acid or protein fragments, and can drift in time to give birth to a hybrid chimeric virus that dissembles parents. Now, it is frightening to imagine that bats, the primordial hosts of CoVs can shelter versatile viral entities at a time. These viruses can mutate, recombine, merge or variate their genetic materials and can render re-emergence of new variants with unknown animosity which is hard to imagine but unfortunately possible. This possibility triggers the probability of recurrence of pandemics in upcoming days, and sirens humanity to get ready to adapt to new plaintive normality (34, 35).

***Thymoquinone: A natural product with therapeutic potential***

**Figure 1:**
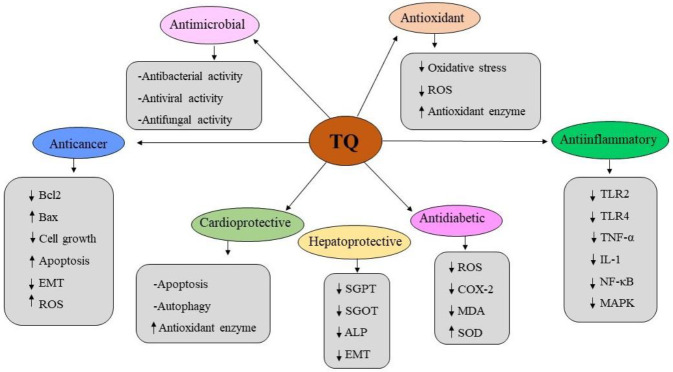
Protective effects of thymoquinone

As a natural phytochemical, TQ has biological activities including antineoplastic, antioxidant, anti-inflammatory, antimicrobial, analgesic, and immunomodulatory effects *in vitro* and *in vivo* (7). A summary of the protective effects of TQ has been presented in Figure 1. TQ has received extensive attention due to its remarkable anticancer activity. The continuous proliferation of cancer cells may indicate the early stage of cancer. Several pieces of evidence suggested that TQ depresses the proliferation, invasion, and metastasis of cancer cells by interfering with different cell signaling pathways (7, 8). TQ has been reported to play an essential role in cervical cancer cells by interfering with the EMT signaling pathway through reduction of the expression of Twist1 and Zeb1 (8, 36). TQ could reverse epigenetic alterations, such as histone acetylation and deacetylation, DNA methylation and demethylation, which can lead to inhibition of gene expression and tumor development (37). TQ can also stimulate cell proliferation and migration by regulating the balance of cytokines and growth factors. Studies have reported that TQ can promote wound healing in diabetic patients through reduction of ROS levels and accelerate cell proliferation and metastasis. In immune system diseases, TQ can affect the production of T cells, B cells, IFNs, ILs, TNF, TGF, and other cytokines by targeting different cell signaling pathways, such as JAK-STAT and PI3K/Akt. TQ is a potent superoxide radical scavenger, which has antioxidant activity against cardiovascular disease (38-40).

Interestingly, TQ also can induce oxidative stress contributing to its antibacterial effect. TQ showed antibacterial effects via up-regulating the expression of ROS which leads to oxidative stress, cell death, and biofilm inhibition activity towards both gram-negative and gram-positive bacteria (41). Although TQ has a wide range of effects and is effective for a variety of diseases, there are not many TQ drugs for clinical treatment, mainly due to its poor solubility, inadequate bioavailability, and easy degradation. The application of TQ inside lipid-nanosystems can improve these deficiencies by protecting the drug, targeting the drug to the organ and thereby, releasing the drug in a controlled manner (42). It is necessary to improve bioavailability for effective application of TQ in therapeutic strategies.

***Thymoquinone and infectious diseases***

**Figure 2:**
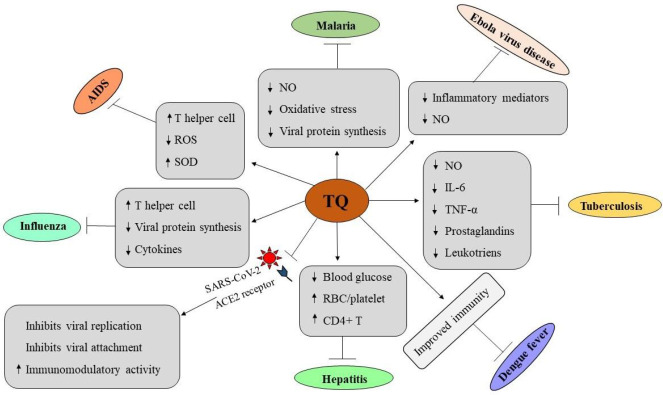
Therapeutic roles of thymoquinone in infectious diseases

TQ can be used as an alternative antimicrobial drug against infectious diseases. TQ has a broad antimicrobial spectrum including Gram-positive bacteria, Gram-negative bacteria, viruses, parasites, Schistosoma, and fungi (43, 44). It showed antimicrobial activity by targeting the intracellular locations of the pathogens. It can improve efficacy versus toxicity and may overcome the drug resistance problem by combining with different conventional chemotherapeutics (10, 15). Hereunder, we discuss the role of TQ in different infectious diseases (summarized in Figure 2 and Table 2). 

*Thymoquinone and recent pandemics caused by SARS-CoV and SARS-CoV-2*

Both SARS-CoV and SARS-CoV-2 are enveloped, single-stranded, positive-sense RNA viruses and usually infect epithelial cells of respiratory organs. Like SARS-CoV, SARS-CoV-2 also uses ACE2 receptors for its entry into the host cells. Additionally, SARS-CoV-2 uses another host cell receptor known as TMPRSS2 (type 2 transmembrane serine protease). The spike protein (S) of SARS-CoV-2 binds strongly with ACE2 receptors and TMPRSS2 to infect host cells. S protein of SARS-CoV-2 has a comparatively higher receptor binding affinity than other coronaviruses. Once inside the host cells, SARS-CoV/SARS-CoV-2 can inhibit the host anti-viral responses that lead to the viral RNA replication and produce cytokine storm syndrome such as increases the production of pro-inflammatory cytokines (e.g., IL-1β, IL-6, TNF-α, and IFN-γ) and mainly affects the respiratory organ cells (45, 46). Interestingly, a recent study suggested that ACE2 expression in epithelial cells is directly correlated with pro-inflammatory cytokine expression in SARS-CoV-2 patients (47). Additionally, it has been found to affect pathogen-associated molecular patterns (PAMPs) structure, reduce RNA sensors in the cytosol, inhibit TLR signaling pathways and TNF receptor-associated factors (TRAF3 and 6), as well as transcription factors such as nuclear factor kappa B (NF-κB), interferon regulatory factors (IRF3 and IRF7), and signal transducer and activator of transcription (STAT) protein (47, 48). However, the exact mechanism of SARS-CoV/2 and cytokine storms syndrome is still under investigation. 

To date, several treatment approaches have been applied to treat SARS-CoV-2 but these are not well established. Natural products may become a potential therapeutic target to treat viral diseases for their prodigious anti-viral mechanism (48). TQ is among the studied natural products for its anti-inflammatory properties against various infectious diseases, including cancer and SARS-CoV/2 (10, 37). It is reported that TQ might interfere with SARS-CoV-2 attachment to the host cell by binding with the heat shock protein A5 (HSPA5) wherein HSPA5 is reported as a binding site of SARS-CoV-2 spike protein (49). An *in silico* study revealed that TQ used a similar pathway like SARS-CoV-2 infection and effectively bound with the ACE2 receptor (50). Notably, TQ and Zinc combination has been proven to play an important role in improving innate and adaptive immunity against viral infections as well as SARS-CoV-2 (51). Xu *et al*. suggested TQ as a potential broad-spectrum inhibitor of coronaviruses that may inhibit viral replication and attachment to host cell receptors (52). TQ might also be effective in reducing SARS-CoV-2 infection severity and controlling the secondary infections in COVID-19 patients by activating anti-inflammatory, immune-regulatory, and antioxidant activities along with down-regulating ACE2 receptors. Therefore, TQ may become a potential approach for developing a drug against viral infection, especially SARS-CoV or SARS-CoV-2 for their prodigious anti-viral effects that inhibit viral attachment and immunomodulatory functions (53). Ulasli *et al*. (54) confirmed that *N.* *sativa* extracts can suppress coronavirus load by increasing the induction of IL-8. Supplementation with *N.* *sativa* oil or its active compounds may also be beneficial through improving comorbidity situations that increase the severity of SARS-CoV-2 infection in patients (55). Recently, a phase III clinical trial is under investigation in which the patients have been administered honey and *N.* *sativa* against SARS-Cov-2 infection (ClinicalTrials.gov, NCT04347382). This trial may help to decrease the complications rate of SARS-Cov-2 infection (56). No reports have been found on TQ’s effectiveness against MERS.

*Thymoquinone and tuberculosis*

Tuberculosis (TB) is caused by the bacteria *Mycobacterium tuberculosis* which affects one-third of the world’s population. Increasing resistance to chemotherapeutic drugs has become a disease resurgence threat and it continues to cause considerable mortality worldwide. Moreover, currently available TB drugs showed numerous side effects, like headache, nausea, and gastrointestinal discomfort. For this, the development of new drugs with improved efficacy has become essential. No new drugs have been developed with complete efficiency in the past 30 years for TB. It is necessary to identify a new anti-TB agent from medicinal plant/natural product with minimal or no side effects (57, 58). 

Some potent bioactive compounds including TQ, piperitone, alantolactone, octyl acetate, 1,8-cineole, α-verbenol, citral b, and α-pinene have been collected from plant species of the Middle East and North Africa (MENA) region. These compounds have been considered as potential sources of novel drugs against TB for their ability to kill the *Mycobacterium* species by affecting the permeability of microbial plasma membranes (58). One study showed that TQ represents a prospective treatment option to combat *M. tuberculosis* infection where mycobacteria-induced nitric oxide (NO) production and pro-inflammatory responses were investigated in *M. tuberculosis* (MTB)-infected Type II human alveolar and human myeloid cell lines. In this study, TQ inhibited the replication of intracellular *M. tuberculosis* strain H37Rv and extensively drug-resistant tuberculosis (XDR-TB) in mouse macrophages. TQ also reduced the production of MTB-induced NO and pro-inflammatory cytokines such as IL-6 and TNF-α in different cell lines (57). Another study also reported that TQ with MIC of 20 µg/ml showed anti-*M. Tuberculosis *activity *in vitro *through suppression of inflammatory mediators such as prostaglandins and leukotrienes (59). Dey *et al*. (60) investigated the antitubercular activity of TQ against multi-drug resistant clinical isolates. They found that TQ exhibited activity against both drug-sensitive and drug-resistant *M. tuberculosis *(lowest MIC of 0.25 µg/ml) by specifically targeting the intracellular locations of the pathogens. TQ can be a promising protective agent in the maintenance of normal hepatic function during treatment with anti-tuberculosis drugs (ATD), as hepatotoxicity or liver damage is the most serious adverse effect of ATD that interrupts the successful completion of TB treatment (61). 

*Thymoquinone and influenza*

Very few studies suggested that TQ has antiviral efficacy against the influenza virus. A study (62) determined the effects of *N. Sativa* seeds on immune responsiveness and pathogenesis of H9N2 avian influenza virus (AIV) in turkeys. *N.* *sativa* seeds can protect poultry from harmful effects of influenza infections by increasing T helper and cytotoxic T cells, enhancing expression of interferon as well as decreasing pro-inflammatory cytokines and viral proteins. 

*Thymoquinone and Dengue*

Dengue fever is a mosquito-borne disease that has become a serious public health concern worldwide. Dengue virus (DENV) infection spreads among humans mainly by the bite of infected *Aedes aegypti*. Millions of people have been affected pervasively in the tropical and sub-tropical states, particularly in the municipal and sub-municipal zones. Almost 400 million people have been infected universally with DENV per year (63). However, proper treatment of DENV infections is as yet unknown. Recently, a tetravalent dengue vaccine (CYD-TDV, Dengvaxia) has been advanced by Sanofi-Pasteur, but its efficacy is not proven. For this, the vaccine has been approved only for emergency use in some pervasive states (64).

In contrast, natural products can be a novel strategy due to their structural and chemical components. Interestingly, the secondary metabolites obtained from natural products have been reported to have antiviral actions (64, 65). Therefore, TQ has been explored as an immune enhancer against dengue fever. Studies reported that the use of TQ may counter mosquito-borne diseases by stimulating the immuno-control approach to limit vector reproduction in the affected persons. Further investigation should be needed to approve TQ as a non-costly and non-toxic immune enhancer in the battle against mosquito-borne diseases (65).

*Thymoquinone and Ebola*

Ebola Virus Disease (EVD) is a deadly viral disease that spreads through infected people and animals. It has severely aﬀected the areas of central Africa and sub-Saharan Africa, principally Gabon, Congo, Uganda, and Sudan. In 2014, approximately 11,325 people have passed away due to EVD (66). For this reason, WHO stated this outbreak as a public health emergency of global alarm (67). 

Although effective treatment of EVD is yet mysterious, in quite a few countries, some investigational drugs are under clinical trials. Up to date, FDA has not certified any drug for global EVD use . Evaluation of natural products (including TQ) for the treatment of EVD may be recognized as a progressed drug, as TQ could be a medicinal nutrition or nutritional supplement for treating EVD (67, 68). TQ has been reported to inhibit the lipopolysaccharide-induced inflammatory processes by suppressing the activities of potent inflammatory mediators along with reducing the concentration of NO that plays a major role in the pathogenesis of EVD (66). However, more experiments are needed to use TQ against EVD clinically.

*Thymoquinone and Zika*

Zika fever also occurs by the bite of a diseased mosquito of the *Aedes* genus like *A. Aegypti*. Body fluids and sexual activities are another way of transmission of the Zika virus (69). The signs and symptoms of Zika fever are almost similar to dengue fever (WHO, 2016). WHO declared Zika fever as the public health emergency of global alarm due to its outbreak in February 2016. During this outbreak, the Zika virus spread through about 38 zones globally (70). 

But, accurate treatment of Zika fever is still unknown. Studies suggested that some natural compounds such as terrestrial or marine-related compounds could show low toxicity and great efficacy in the case of Zika fever treatment during a particularly serious outbreak (71). Moreover, TQ is not proven against Zika fever. Although further experimental investigations of TQ on Zika fever may open the potential door. 

*Thymoquinone and hepatitis*

Hepatitis is caused by viral infections only in humans and is extremely transmissible. Mainly five types of viruses are responsible for the infections, e.g., hepatitis A virus (HAV), hepatitis B virus (HBV), hepatitis C virus (HCV), hepatitis D virus (HDV), and hepatitis E virus (HEV). It has been already declared as a major public health alarm globally. Basically, spread of hepatitis is done by parenteral or mucosal exposure to diseased blood and through body fluids, namely semen, and vaginal fluid, etc. (72, 73). According to WHO, approximately 1.3 million people died due to HBV or HCV in 2015. WHO also stated that around 2 billion people were affected by HBV, 185 million people by HCV, and 20 million people by HEV (73). 

However, proper treatment of hepatitis is now available. For example, pegylated interferon-α (PEG-IFNα) and ribavirin are the only official drugs for HCV treatment without major or light side effects. Remarkably, natural medicine is also offered for this disease. Recently, an important report has informed that TQ treatment showed enhancement of RBC and platelet counts, reduction of HCV viral load, amplification of total antioxidant movement along with albumin levels, reduction of blood glucose levels, and enhancement of lower-limb edema in HCV patients (74). Currently, hepatic ailment induced by hepatitis is also being treated by TQ. TQ augmented the levels of serum interferon-γ (IFN-γ), numbers of CD4+ T cells and macrophages, and inhibited the growth of fatty acid in the hepatocytes. TQ can repress the production of inflammatory mediators in the liver through inhibition of the PI3K/Akt/NF-κB signaling pathway (75, 76). Administration of ethanolic extract of *N.* *sativa* in HCV patients was found to enhance α-fetoprotein and other liver function parameters as well as decrease viral load significantly. A study conducted on HCV patients in Egypt demonstrated the beneficial effect of the mixture of ethanolic extracts of NS and *Zingiber officinale*. In that study, patients were treated with capsules containing 500 mg of this mixture twice daily for one month, and improved liver function was observed along with decreased viral load (77). 

*Thymoquinone and malaria*

A life-threatening ailment named malaria is spread by *Plasmodium* parasites according to the updated World Malaria Report. Its spread occurs with the bites of diseased female *Anopheles* mosquitoes, wherein children aged less than 5 years are in a dangerous situation. Globally, around 229 million people were infected with malaria and 409,000 people died in 2019 (78). It is proposed as a major public health concern due to morbidity and mortality worldwide (79). Anti-malarial drugs are open access but their resistance, especially *Plasmodium falciparum* resistance, is one of the major challenges against malarial mechanisms. Thus, for the reason of ineffectiveness of antimalarial drugs in terms of resistance, malaria-related mortality has enhanced in the last 3 decades (80). 

From the previous studies, a very productive approach for medicinal chemistry and drug design has been opened by the hybridization of natural products. So, its enhancement of pharmacological natures like augmented biological efficacy, diminished side effects, lesser toxicity, and improved bioavailability have been examined (81). TQ may inhibit NO production in macrophages, can counteract oxidative damage, and can produce antimalarial properties by blocking protein synthesis in *Plasmodium *parasites (82). TQ showed anti-proliferative activity against *P. falciparum* in malaria. Besides, a combined dose of TQ and artemisinin has also been explored for the therapeutic activity against *P. falciparum *(81). A study (83) reported that dietary supplementation of *N.* *sativa* seed and oil extract in combination with chloroquine could be beneficial in the treatment of malaria. Another experiment also suggested that *N. sativa* extract has significant anti-malarial action in malarial treatment. So, ongoing antimalarial resistance difficulties might be overwhelmed by the magical seeds of this prospective plant (80). 

*Thymoquinone and HIV*

The human immunodeficiency virus (HIV) causes a chronic and possibly life-threatening disease called acquired immunodeficiency syndrome (AIDS). It is also a major public health concern worldwide. Approximately 38 million (36.2 million adults and 1.8 million children) people were infected with HIV and 690,000 people had died. The spread of HIV can happen from diseased people with body fluids like blood, breast milk, semen, vaginal secretions, etc. (84, 85). 

Bone marrow transplantation, some vaccines, cytotoxic agents, and early instigation of HAART (highly active antiretroviral therapy) are probable therapeutic mediators for HIV patients. All are regarded as ineffective solutions, with bone marrow transplantation as costly and risky option. Moreover, prolonged use of HAART with the inclusion of HIV-1 protease inhibitors is associated with insulin resistance syndrome. Exposure to several different HIV-1 protease inhibitors decreases glucose-stimulated insulin secretion from pancreatic beta-cells, significantly increased reactive oxygen species (ROS) generation, and suppressed superoxide dismutase (SOD) levels. TQ may be used as a potential therapeutic agent or dietary supplement to prevent the deleterious effects observed in HAART-treated patients (86). Additionally, the combined treatment of TQ and honey is unequivocally recognized which stimulates escalation of CD4 count and reduction of viral load in HIV patients. TQ can also increase T helper cells and other leucocytes (87, 88). Moreover, a sustained seroreversion was reported in a 27-year-old HIV-positive pregnant woman who was not eligible for antiretroviral therapy, took 10 ml of *N*. *sativa* concoction (60% of *N*. *sativa* seeds and 40% of honey) three times daily for a year. Serology assessments was repeated for 10 years, HIV infection became negative with undetectable viral load and CD4 count not less than 750 cells/μl (89). In a study, a 46-years old HIV-positive patient recovered entirely after treating with 10 ml of the black seed of *N. sativa* twice daily for 6 months (90). So, more experiments may confirm TQ as an encouraging remedy in the cases of HIV patients (10). 

***Facts and reality***

It has become a real challenge to search and specify the effective compounds from natural products for humans. Moreover, developing natural compounds into pharmaceuticals remains difficult yet due to poor biopharmaceutical properties like solubility, bioavailability, stability, and biological reproducibility (1, 2, 91). Pharmaceutical companies need to improve the stringency of trial policy and higher safety requirements for drug licensing (91, 92). Several studies have confirmed that TQ possesses promising activities against infectious diseases through multiple approaches both *in vitro* and *in vivo*. However, these studies are still in the preclinical stages, and it is a long way to go to get the complete clinical benefits of TQ (91). More extensive *in vivo *studies on appropriate animal models and clinical trials are required to explore the antimicrobial potential of TQ for developing a drug against infectious diseases along with investigation of their safety in humans. Pharmacokinetic studies of TQ should also be conducted to clarify pharmacokinetic profiles (e.g., absorption, distribution, metabolism, and excretion parameters). In near future, TQ with more novel drug delivery carrier systems, improved bioavailability, enhanced stability, reduced dose, more efficacy, and diminished side effects are expected (10, 11, 93).

**Table 1:** Recent pandemics and risk of viral diseases in 21^st^ century

**Infectious diseases**	**Causative virus**	**Year of pandemic**	**First reported in**	**Transmission vector**
SARS	SARS-CoV	2003	China	Horseshoe bat
H1N1	Influenza A (A/H1N1)	2009	Mexico	Pig
MERS	MERS-CoV	2012	Middle East	Camel
Chikungunya	CHIKV	2014	America	Mosquito
Zika fever	Zika	2015	America, Asia, Africa	Mosquito
EVD	Ebola	2014 to 2015	African countries	Fruit bat
COVID-19	SARS-CoV-2	2019-present	China	Bat

TQ has potential protective effects against many diseases including anticancer, antioxidant, anti-inflammatory, antidiabetic, antimicrobial, cardioprotective, and hepatoprotective effects

TQ possesses antimicrobial properties and can be an important therapeutic option against infectious diseases. It can regulate the secretion of disease-causing agents by targeting important cell signaling pathways

**Table 2:** Role of thymoquinone in infectious diseases

**Disease**	**Experimental model**	**Effects**	**Reference**
COVID19	HEK293 cells	Inhibits viral replication and attachment to host cell receptors; may suppress viral load and reduce the severity of viral infection	[51-53]
TB	Type II human alveolar and human myeloid cell lines	Reduces the production of NO and pro-inflammatory cytokines	[57,59]
Influenza	Turkeys	Increases T helper and cytotoxic T cells, enhances expression of interferon as well as decreasing pro-inflammatory cytokines and viral proteins	[62]
Dengue fever	Patients	Acts as immune enhancer	[65]
Ebola virus disease	Glial cells	Inhibits the lipopolysaccharide‑induced inflammatory processes	[66]
Hepatitis	HCV patients	Reduces viral load, increases numbers of CD4+ T cells and macrophages	[75-77]
Malaria	Mice	Inhibits NO production in macrophages and can counteract the oxidative damage	[83]
AIDS	HIV positive patients	Reduces viral load, increases numbers of CD4+ T cells and macrophages	[88,89]

## Conclusion

Infectious diseases have become a threat to modern human lives due to antimicrobial-resistant infections. With the evidence of recent pandemics, it is warned that more deadly pandemics can come and become more lethal in the future. So, it is necessary to develop new effective treatments that circumvent microbial resistance. TQ can be important preventive and therapeutic options against infectious diseases due to its multi-targeting and non-toxic nature. It possesses many beneficial pharmacological properties and can inhibit important cell signaling pathways which may regulate the secretion of disease-causing agents. However, there have been limited clinical trials employing TQ for the treatment of infectious diseases due to poor solubility, bioavailability, and sensitivity. To overcome the limitations of TQ, it is necessary to incorporate TQ in drug delivery systems. More laboratory and clinical studies are required for better understanding the molecular mechanism of TQ action to explore the therapeutic use of TQ in the treatment of microbial infectious diseases with limited side effects and a more convenient drug delivery system.
